# SUPPORT FOR FAMILY CAREGIVERS: WORK STRAIN AND THE IMPACTS OF FORMAL AND INFORMAL SUPPORT

**DOI:** 10.1093/geroni/igad104.0467

**Published:** 2023-12-21

**Authors:** Joseph Svec, Natasha Nemmers, Jeong Eun Lee

**Affiliations:** Saint Joseph’s University, New York City, New York, United States; Wayne State University Institute of Gerontology, Detroit, Michigan, United States; Iowa State University, Ames, Iowa, United States

## Abstract

In the US, caregiver burnout has significant mental and physical ramifications for an estimated 40 million unpaid caregivers. In addition to higher risks of depression, fatigue, and anxiety, caregivers are often squeezed in the already tenuous balance between work and life. This study seeks to assess whether and to what extent the mental health consequences of caregiver work strain is ameliorated by the presence of additional (informal) family caregivers and formal service use. This study utilizes data provided by the National Study of Caregiving (NSOC) data which is linked with care-recipient information from Health and Aging Trends Study (NHATS) data. We analyze mental health outcomes for caregivers who were present in years 2015 and 2017 (N = 281) using lagged linear regression techniques. We estimate associations of work-strain with mental health issues as well as the moderating effects of informal support (the number of additional caregivers) and utilization of formal support (such as paid service support, formal training, and Medicare funding). Preliminary analyses indicate that the additional informal support mitigates the effect of work strain on mental health. However, formal support corresponds with positive mental health outcomes in the absence of work strain and poorer mental health outcomes when the caregiver experiences work strain. One possible explanation is that formal support is more likely to be used when caregiver burden and isolation is higher, highlighting the importance of timing in service utilization as well as the importance of social and informal resources for employed caregivers.

